# Obesity and adiposity promote the development of non-suppurative otitis media: a Mendelian randomization study

**DOI:** 10.3389/fmed.2024.1422786

**Published:** 2024-07-10

**Authors:** Xin Yan, Suhua Chen

**Affiliations:** ^1^Department of Otolaryngology, Shaoxing People’s Hospital, Shaoxing, China; ^2^Department of Pharmacy, Shaoxing People’s Hospital, Shaoxing, China

**Keywords:** obesity, lipid, Mendelian randomization, high-density lipoprotein, apolipoprotein A1

## Abstract

**Background:**

Observational studies have found that obesity is associated with the development of non-suppurative otitis media (NSOM), but the causality and pathogenesis are unclear. This study aimed to investigate the association between obesity, lipid metabolism, and NSOM at the genetic level.

**Methods:**

We performed a bidirectional two-sample Mendelian randomization (MR) study to examine the causal relationship between obesity, lipid metabolism-related factors, and NSOM by using the datasets obtained from the IEU Open genome-wide association studies (GWAS) Project. Furthermore, a multivariate MR (MVMR) analysis on lipid indicators was conducted to validate the results. We then used obesity or body mass index (BMI) as the exposure and NSOM as the outcome to search for possible mediators in lipids and adipokines.

**Results:**

Using NSOM as the outcome, we found nine positive exposure results related to obesity and lipid metabolism. Among them, obesity, BMI, body fat percentage, waist circumference, hip circumference, and resistin were risk factors, while apolipoprotein A1 (apoA1), high-density lipoprotein cholesterol (HDL-C), and nerve growth factor (NGF) were protective factors. Then, we used the obesity and lipid metabolism-related factors as outcomes and NSOM as the exposure to perform the MR analysis, which failed to obtain positive results. In the MVMR analysis, we found that HDL cholesterol and apoA1 remained causally associated with NSOM after correction for other potential confounders. Simultaneously, when obesity or BMI was used as the exposure and NSOM as the outcome, HDL cholesterol or apoA1 served as mediators through a two-step MR analysis. The MR analysis for mediation, obesity, and BMI reduced the production of HDL or apoA1, which served as protective factors affecting the development of NSOM.

**Conclusion:**

At the genetic level, obesity and adiposity may promote the development of NSOM, while NSOM has no effect on obesity and adiposity. Obesity can also encourage the progress of NSOM by reducing HDL cholesterol/apoA1. Resistin may be a potential risk factor for NSOM, whereas NGF may be a potential protective factor.

## Introduction

Non-suppurative otitis media (NSOM), also known as secretory otitis media, exudative otitis media, serous otitis media, catarrhal otitis media, and tympanic cavity effusion, is a common inflammatory disease of the middle ear. It is usually caused by poor Eustachian tube function and is characterized by otitis media effusion and hearing loss. NSOM is most common in children, and its incidence rate is very high. It usually develops between 6 months and 4 years old (1). In total, 50–90% of children under the age of 5 have a history of NSOM (2). It is also one of the leading causes of hearing loss in children and even has an impact on their intelligence and language development.

Obesity is a global issue of great concern, and it is becoming more serious as living standards rise (3). Obesity has been shown to cause a variety of diseases, including cardiovascular (4) and cerebrovascular disease (5), diabetes (6), obstructive sleep apnea syndrome (7), and metabolic disorders (8). Observational studies have shown that NSOM may be associated with obesity, most of which is concentrated in the pediatric population (9, 10). In addition, observational studies have also found that a high-fat diet rather than obesity is associated with NSOM, and a high-fat diet is a confounding factor between obesity and NSOM (11). At present, the relationship between the two is still not very precise, the mechanism of action and the causal relationship are not clear, and the observational research is easily influenced by confounding factors.

Mendelian randomization (MR) can use genetic instrumental variables to test the potential causal relationship between exposures and outcomes. Because genetic variation occurs randomly and is not influenced by external environmental factors, MR analysis minimizes potential unpredictable confounding factors and compensates for the shortcomings of observational research (12). Cao et al. (13) used MR methods to demonstrate that childhood body mass index is a risk factor for the development of NSOM in children. Considering the correlation between lipid metabolism-related factors and obesity, we also included them in the study and used MR methods to analyze the relationship between obesity, obesity indicators, lipids, adipokines, and NSOM, to gain a preliminary understanding of the disease pathogenesis.

## Methods

The exposure and outcome data for MR analysis were collected from the datasets of the IEU Open GWAS Project, and the data on obesity and lipid metabolism were divided into four categories: obesity (phenotype of obesity), obesity indicators, lipids, and adipokines. Obesity indicators include body mass index (BMI), body fat percentage, waist circumference, and hip circumference; lipids include total cholesterol, low-density lipoprotein cholesterol (LDL cholesterol, LDL-C), high-density lipoprotein cholesterol (HDL cholesterol, HDL-C), triglycerides, apolipoprotein A1 (apoA1), and apolipoprotein B (apoB); adipokines include adiponectin, resistin, leptin, agouti-related protein, and nerve growth factor (NGF). There were a total of 16 obesity and lipid metabolism-related GWAS datasets as exposure data. The outcome data were NSOM. The details of the GWAS datasets from the IEU website are shown in Table 1.

**Table 1:** Detailed information on the dataset used in this article from the IEU website.

Category	Traits	PMID	Year	Sample Size	Number of SNPs	Gender	Population
Exposures (obesity)	Obesity	22,484,627	2012	13,848	2,430,514	NA	European
Exposures (obesity indicators)	Body mass index	25,673,413	2015	236,781	2,529,499	NA	European
	Body fat percentage	/	2017	331,117	10,894,596	Males and Females	European
	Waist circumference	34,017,140	2021	407,661	10,783,687	NA	European
	Hip circumference	25,673,412	2015	127,997	2,444,355	Females	European
Exposures (lipids)	Total cholesterol	34,226,706	2021	437,878	4,232,052	NA	European
	LDL cholesterol	34,594,039	2021	343,621	19,037,976	NA	European
	HDL cholesterol	24,097,068	2013	94,595	2,418,527	NA	European
	Triglycerides	32,203,549	2020	441,016	12,321,875	Males and Females	European
	Apolipoprotein A1	35,213,538	2022	115,082	11,590,399	NA	European
	Apolipoprotein B	34,226,706	2021	435,744	4,231,412	NA	European
Exposures (adipokines)	Adiponectin	22,479,202	2012	39,883	2,675,209	Males and Females	Mixed
	Resistin	33,067,605	2020	21,758	13,138,697	NA	European
	Leptin	32,917,775	2020	56,802	231,001	NA	Mixed
	Agouti-related protein	33,067,605	2020	21,758	13,102,571	NA	European
	Nerve growth factor	28,369,058	2018	3,394	5,270,646	Males and Females	European
Outcome	NSOM	/	2021	/	16,380,433	Males and Females	European

GWAS, genome-wide association study; SNP, single nucleotide polymorphisms; LDL, low-density lipoprotein; HDL, high-density lipoprotein; NSOM: non-suppurative otitis media.

To meet the three hypotheses of the MR analysis and minimize the influence of confounding factors, we extracted single nucleotide polymorphisms (SNPs) as instrumental variables that met the following conditions: a clustering window of 10 MB and an r2 cutoff of 0.001. SNPs associated with every trait were extracted at a significance threshold of *p* < 5 e- 8, but if there were few SNPs extracted for Mendelian randomization, we would reanalyze them at *p* < 5e-6 or *P*<5e-5.

In total, 16 datasets related to obesity and lipid metabolism were used as exposures, with NSOM as the outcome. A two-sample MR analysis was performed using MR Egger, weighted media, inverse variance weighted (IVW), simple mode, and weighted mode, in which IVW was used as the main analysis method (referred to as forward MR analysis). If the IVW method produced a *p-*value less than 0.05, a statistically significant causal relationship between exposure and outcome was considered. Additionally, if the odds ratio (OR) >1, risk factors for the development of outcomes were considered; if OR < 1, protective factors were considered. To measure the heterogeneity among SNPs, Cochran’s Q test was employed with the MR Egger and IVW methods, and the MR Egger intercept method was used for pleiotropy testing. If the *p-*value of the IVW method was between 0.04 and 0.05, further validation of the sensitivity of the results would be conducted using the “Leave-one-out” analysis, which can remove each SNP one at a time and track how each SNP affects the combined results.

Then, we used NSOM as the exposure and 16 obesity and lipid metabolism-related datasets as the outcomes for a reverse two-sample MR analysis using the MR Egger and IVW methods. There was a statistically significant causal relationship between the outcome and exposure when using the IVW method, with a *p*-value of <0.05. The subsequent validation methods were the same as the forward MR analysis described above.

Due to the interaction between many lipid indicators, we selectively conducted a multivariate MR (MVMR) analysis on lipids to further verify the reliability of the results.

Finally, we employed a two-step MR analysis using the five methods mentioned above, with IVW as the main method. We used obesity and BMI as exposures and NSOM as the outcome to identify potential mediating variables in lipids and adipokines with a causal relationship to NSOM. Both steps used the method of two-sample MR to avoid confounding factors, and SNPs duplicated in the first step were deleted during the second step of the MR analysis. A mediating effect was considered to exist if the *p-*value of both steps in the MR analysis using the IVW method was less than 0.05. We called the total effect value of exposure to outcome “beta all,” the effect value of the first step of MR analysis of exposure to the mediator “beta1,” the effect value of the second step of MR analysis of mediator to outcome “beta2,” the mediator effect value “beta12”(beta1*beta2), the direct effect value of exposure to outcome “beta_dir”(beta all-beta1*beta2), and the ratio of mediator effect “beta_per”(beta1*beta2/beta all).

All data analyses were processed using R 4.3.2 and related extension packages.

## Results

### Two-sample MR

#### Forward MR analysis

In total, 16 datasets related to obesity and lipid metabolism were used as exposures, with NSOM as the outcome. SNPs associated with all traits were extracted at a significance threshold of *p* < 5 e- 8. There were a total of nine positive factors, namely, obesity, BMI, body fat percentage, waist circumference, hip circumference, HDL cholesterol, apoA1, resistin, and NGF. Among all positive factors, the direction of action in the results using the five methods was also consistent.

#### Obesity (phenotype of obesity)

The IVW analysis revealed a causal relationship between obesity and NSOM (*p* = 0.02); the OR value greater than 1 indicated that obesity was a risk factor for NSOM. The Cochran’s Q *p-*value and the MR Egger intercept *p*-value were both greater than 0.05, indicating that there was no heterogeneity and pleiotropy in the result.

#### Obesity indicators

**Figure 1:**
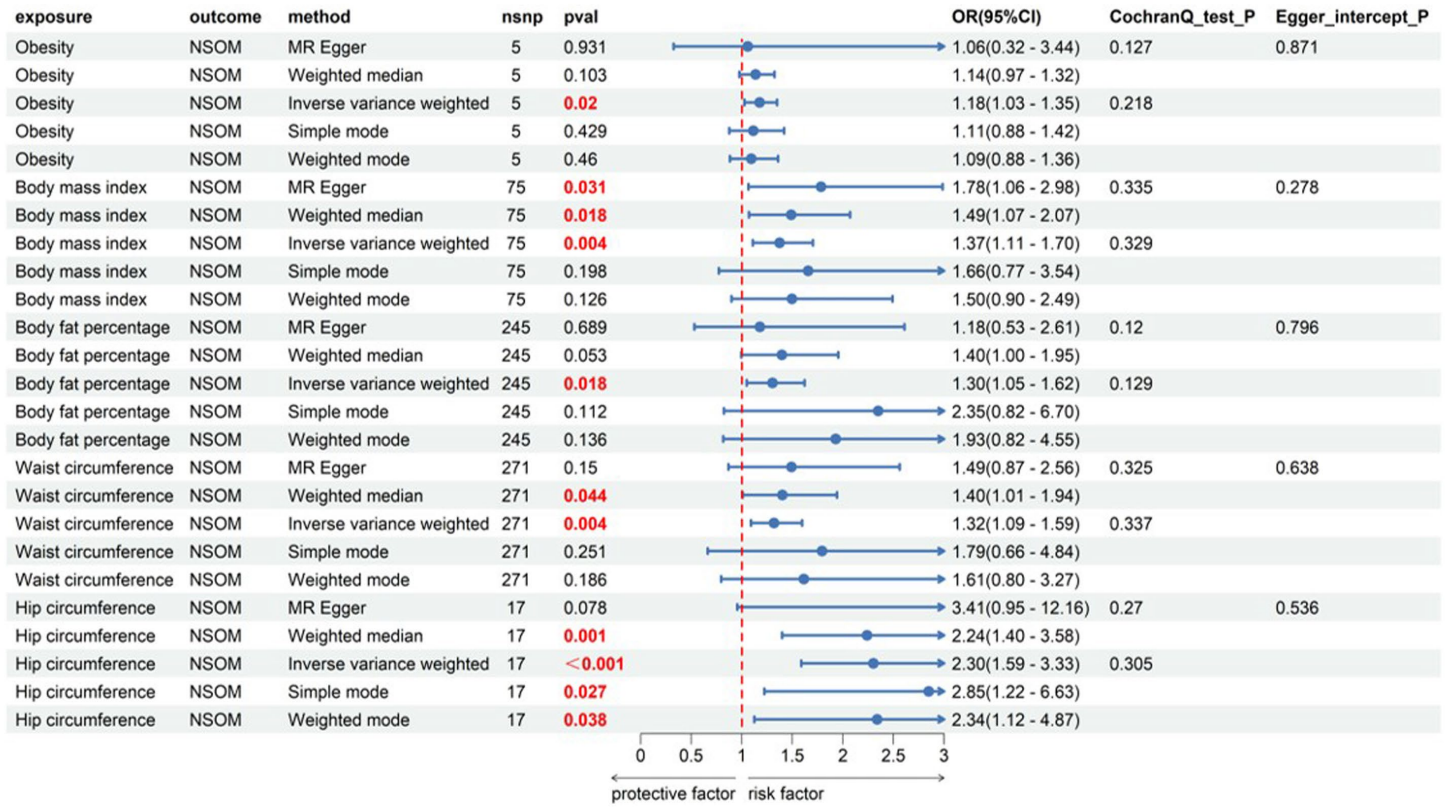
Forward Mendelian randomization analysis data on obesity, obesity indicator, and NSOM. The red text denotes the *p-*value for this trait is less than 0.05. nsnp, number of single nucleotide polymorphisms used as instrumental variables for Mendelian randomization; OR, odds ratio; CI, confidence interval; NSOM, non-suppurative otitis media.

All four obesity indicators were positive exposures, including BMI, body fat percentage, waist circumference, and hip circumference. The OR values for the obesity indicators were all greater than 1, specifically 1.37, 1.30, 1.32, and 2.30, showing that these four indicators were risk factors for the development of NSOM. In the heterogeneity test, all Cochran’s *Q p-*values were greater than 0.05, indicating that there was no heterogeneity in the results. The MR Egger intercept for all dataset results had *p-*values greater than 0.05, indicating that there was no pleiotropy in the data results (Figure 1).

#### Lipids

**Figure 2:**
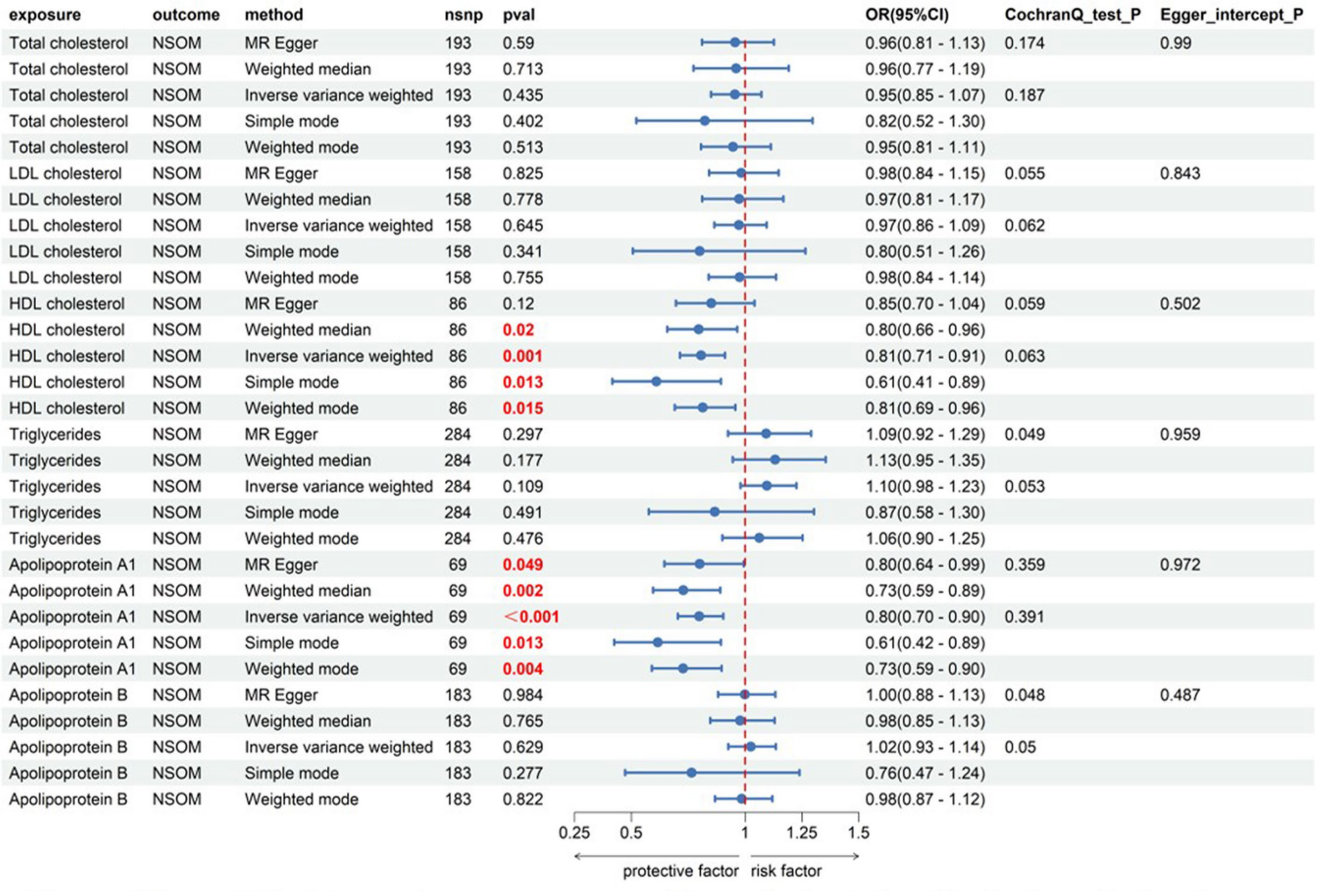
Forward Mendelian randomization analysis data on lipids and NSOM. The red text denotes that the *p-*value for this trait is less than 0.05.nsnp, number of single nucleotide polymorphisms used as instrumental variables for NSOM, non-suppurative otitis media.

Among the six indicators, HDL-C and apoA1 had a causal relationship with the outcome of NSOM, and their OR values were all less than 1, indicating that they were protective factors against the progression of the disease. In the heterogeneity test, all Cochran’s Q *p-*values were greater than 0.05, indicating that there was no heterogeneity in the results. The *p-*values of MR Egger intercept for all results were all greater than 0.05, indicating that there was no horizontal pleiotropy (Figure 2).

#### Adipokines

**Figure 3:**
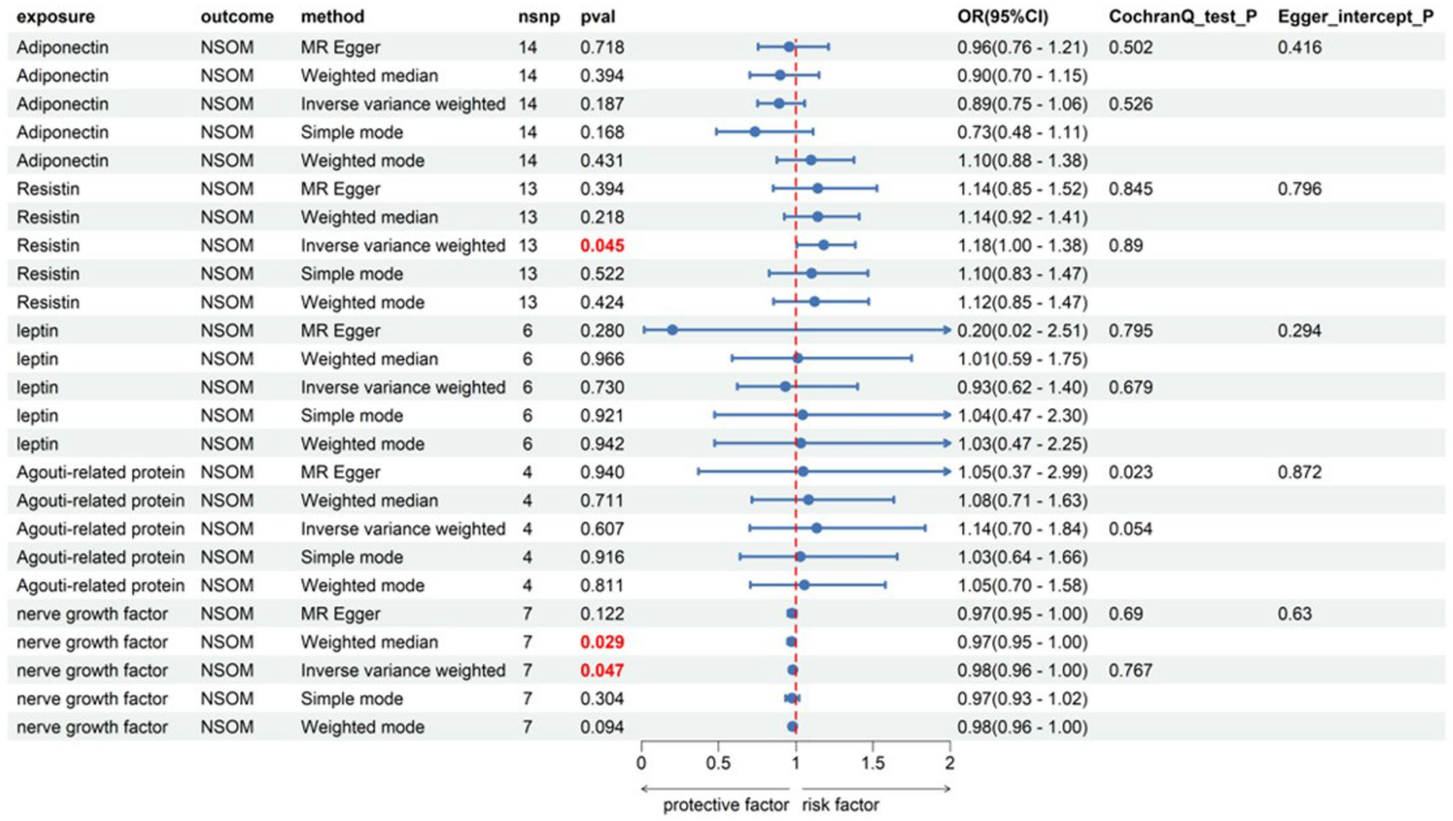
Forward Mendelian randomization analysis data on adipokines and NSOM. The red text denotes the *p-*value for this trait is less than 0.05. nsnp, number of single nucleotide polymorphisms used as instrumental variables for Mendelian randomization; OR, odds ratio; CI, confidence interval; NSOM, non-suppurative otitis media.

**Figure 4:**
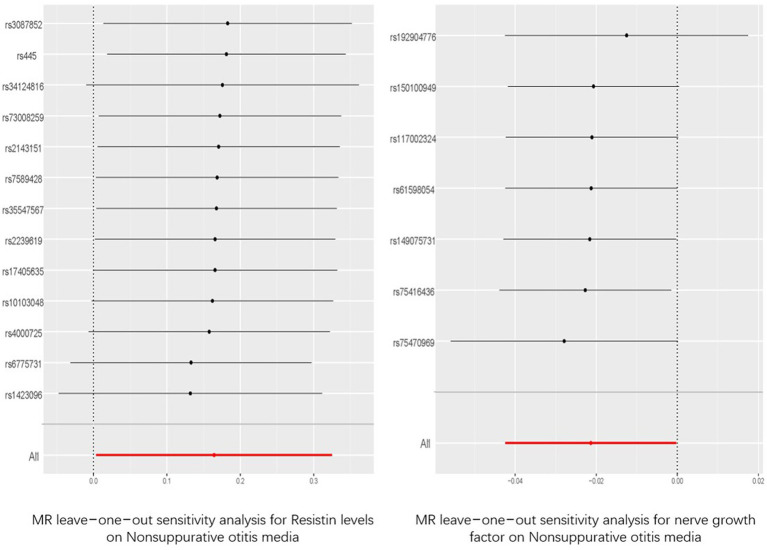
“Leave-one-out” analysis of the causal association of resisitin levels, nerve growth factor and Nonsuppurative otitis media. The 95% CI and causal estimate when each SNP was eliminated individually are shown by the black bars and dots. The fixed-effect IVW method‘s overall estimate and 95% confidence interval are shown by the red dot and bar. CI, confidence interval; SNP, single nucleotide polymorphism; IVW, inverse-variance weighted.

Among the five indicators of adiponectin, the *p-*values of resistin and NGF using the IVW analysis method were less than 0.05, indicating that the two were positive exposures. Resistin, with an OR greater than 1, was identified as a pathogenic factor for the progression of the disease, while NGF, with an OR lower than 1, was considered a protective factor against disease progression. According to the data in Figure 3, heterogeneity and horizontal pleiotropy of the two positive exposures can be excluded. The “Leave-one-out” analysis of susceptibility shows that the MR test was reliable (Figure 4).

#### Reverse Mendelian randomization analysis

Using the 16 obesity- and lipid metabolism-related factors as outcomes and NSOM as the exposure, the MR analysis was performed using the IVW and MR Egger methods, and it was found that all datasets had no positive results. The *p-*values of all IVW analysis results were greater than 0.05. To obtain sufficient instrumental variables, we set the threshold for extracting significant SNPs to *p* < 5e-6, except for acting leptin as the outcome to *p* < 5e-5. Although the *p*-value of Cochran’s *Q* test for several data was less than 0.05, it may be because the data came from different analysis platforms, experiments, and populations. There was no pleiotropy in the data results, as indicated by *p-*values larger than 0.05 for the MR Egger intercept for all dataset outcomes (Supplementary Table S1).

#### MVMR

**Figure 5:**
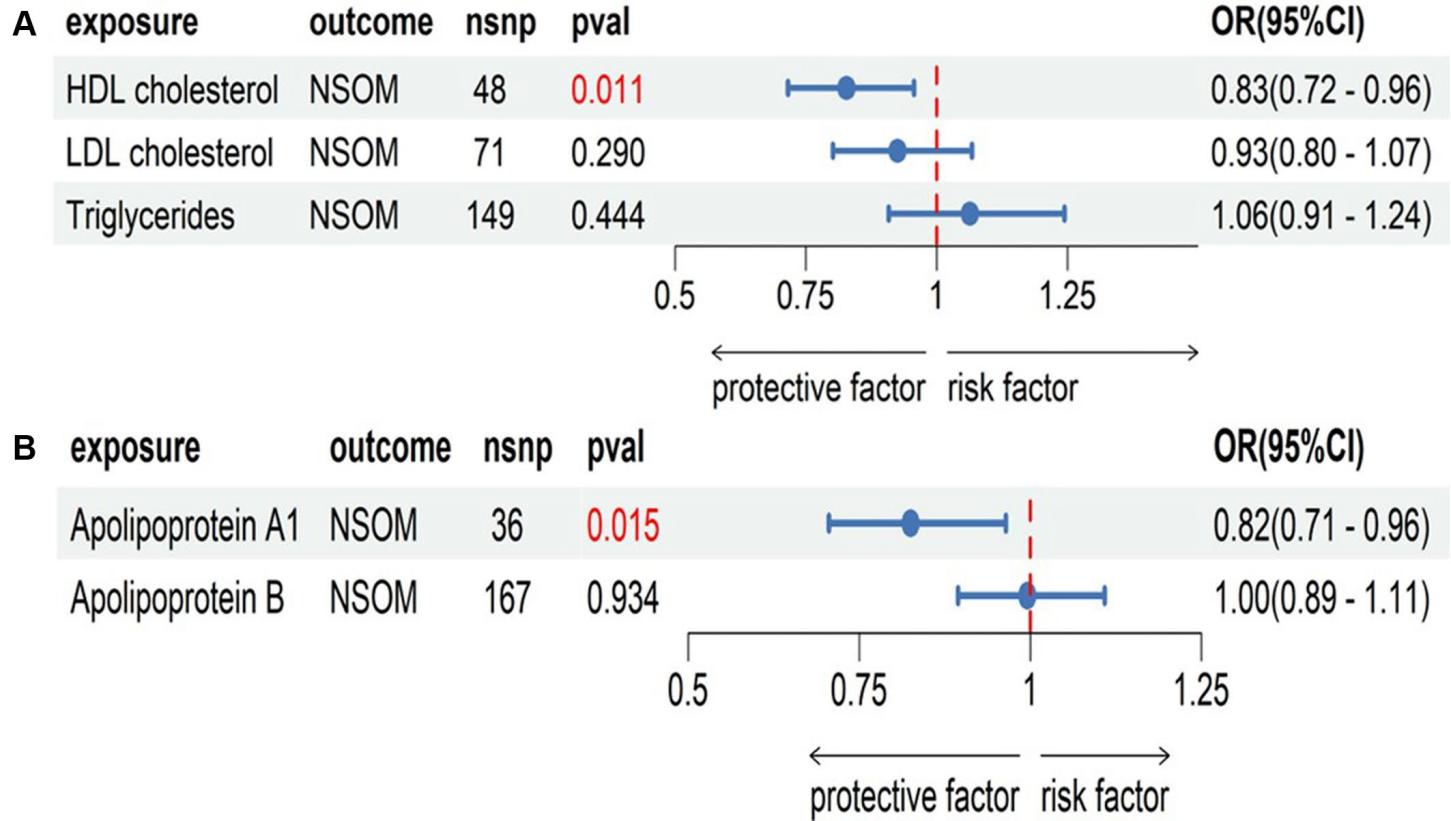
Multivariate Mendelian randomization analysis, **(A)** the group of HDL cholesterol, LDL cholesterol, and triglycerides; **(B)** the group of apolipoprotein Al and apolipoprotein B. The red text denotes that the *p-*value for this trait is less than 0.05. nsnp, the number of single nucleotide polymorphisms used as instrumental variables for NSOM, non-suppurative otitis media.

Combining the positive results obtained in the two-sample MR analysis, we grouped HDL-C, LDL-C, and triglycerides into one group and apoA1 and apoB into another group. By correcting for the effects of LDL-C and triglycerides on NSOM, the causal effect of HDL-C on NSOM remained significant. By correcting for the effects of apoB on NSOM, the causal effect of apoA1 on NSOM also remained significant (Figure 5).

#### Two-step MR

**Figure 6:**
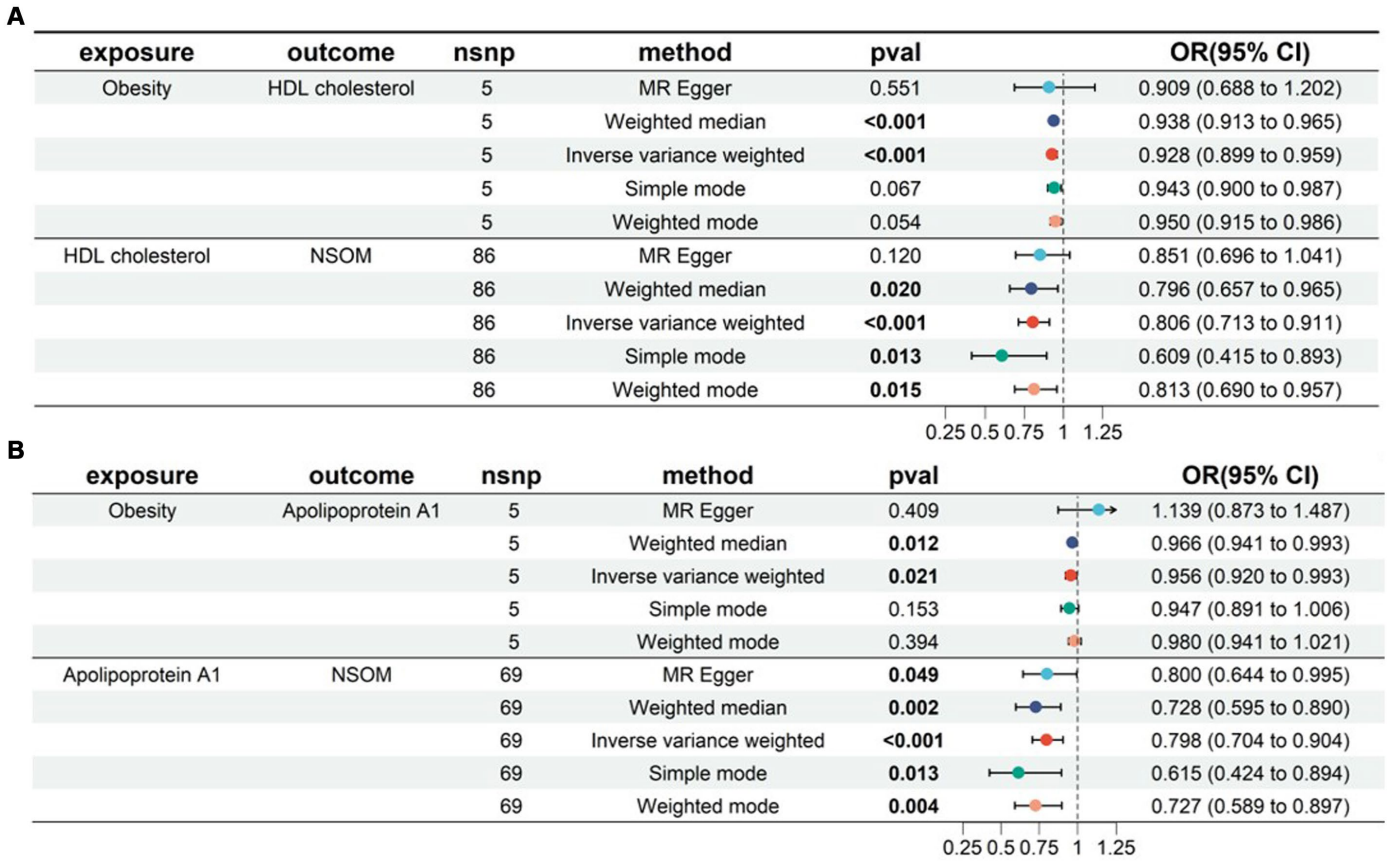
Two-step MR for mediation analysis of obesity as the exposure and NSOM as the outcome. The bold text denotes the *p-*value for this trait is less than 0.05. **(A)** The mediation is HDL cholesterol. **(B)** The mediation is apolipoprotein Al. nsnp: number of single nucleotide polymorphisms used as instrumental variables for Mendelian randomization; OR, odds ratio; CI, confidence interval; NSOM, non-suppurative otitis media.

**Figure 7:**
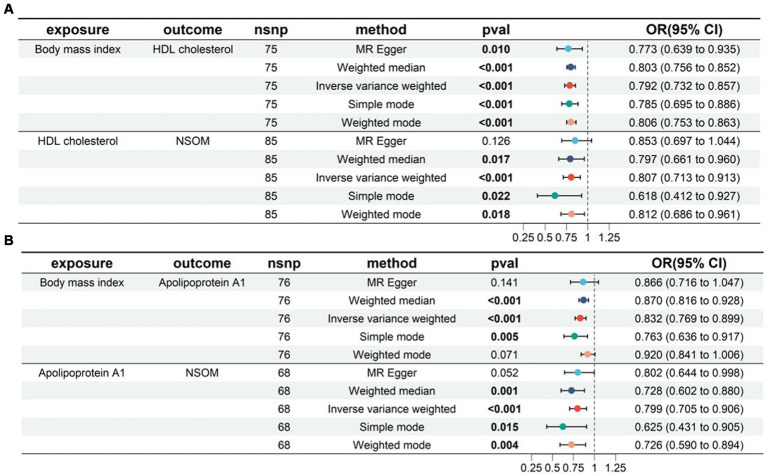
Two-step MR for mediation analysis of body mass index as the exposure and NSOM as the outcome. The bold text denotes that the *p-*value for this trait is less than 0.05. **(A)** The mediation is HDL cholesterol. **(B)** The mediation is apolipoprotein Al. nsnp: number of single nucleotide polymorphisms used as instrumental variables for Mendelian randomization; OR, odds ratio; CI, confidence interval; NSOM, non-suppurative otitis media.

In the two-sample MR analysis, we obtained four lipid metabolism-related factors causally associated with NSOM: HDL-C, apoA1, resistin, and NGF. In the two-step MR analysis, we used these four datasets as a suspected mediator and set the threshold for extracting significant SNPs to *p* < 5e-8. The analysis revealed that HDL and apoA1 could serve as mediators when using obesity as an exposure (Figure 6). The mediation effects were 0.016 and 0.01, respectively, and the percentage of the mediation effects was 0.099 and 0.063, respectively. HDL and apoA1 can also be used as mediators when using the BMI as the exposure (Figure 7); the mediation effects were 0.05 and 0.041, respectively, and the percentage of the mediation effects was 0.158 and 0.13, respectively. The direction of action with the MR Egger method was different from the other four methods when using obesity as the exposure and apoA1 as the outcome; except for the above, the direction of the results using the five methods used for MR was consistent. The *p-*value of the MR Egger intercept was greater than 0.05 for all analyzed procedures, indicating that there was no pleiotropy (Supplementary Table S2) (Table 2).

**Table 2:** The mediating effect of two-step MR mediation analysis.

Exposure	Mediator	Outcome	Beta_all	Beta1	Beta2	Beta12	Beta_dir	Beta_per
Obesity	HDL cholesterol	NSOM	0.162	−0.074	−0.216	0.016	0.146	0.099
Obesity	Apolipoprotein A1	NSOM	0.162	−0.045	−0.226	0.01	0.267	0.063
Body mass index	HDL cholesterol	NSOM	0.317	−0.233	−0.214	0.05	0.267	0.158
Body mass index	Apolipoprotein A1	NSOM	0.317	−0.184	−0.224	0.041	0.276	0.13

nsnp, number of single nucleotide polymorphisms used as instrumental variables for Mendelian randomization. NSOM, non-suppurative otitis media.

## Discussion

In this study, we used an MR method of multi-factors to explore the genetic impact of obesity, obesity indicators, lipids, and adipokines on the risk of NSOM. We aimed to explore the potential factors and pathogenesis of NSOM related to obesity and lipid metabolism and strived to find new treatments, thereby reducing the incidence rate of NSOM and improving its prognosis. We used five methods in MR analysis, namely MR Egger, weighted media, IVW, simple mode, and weighted mode. Although many results showed inconsistent MR estimates, considering the advantage of IVW in maintaining higher estimation accuracy (14), if the *p-*value of IVW was less than 0.05, we believed that there was a statistically significant causal relationship between exposure and outcome. In this MR analysis, a total of nine factors of obesity and lipid metabolism were found to have a causal relationship with the risk of NSOM, which is consistent with the conclusion drawn from observational studies that obesity may affect the development of NSOM and provides a theoretical basis for its related pathogenesis.

This study found that obesity and four obesity-related indicators, namely obesity, BMI, body fat percentage, waist circumference, and hip circumference, all increase the risk of NSOM, indicating that obesity and increased body fat were risk factors for NSOM. In observational studies, the BMI of children with NSOM was significantly higher than that of normal children (9, 10, 15). Kim et al. found that 21.4% of children with NSOM were overweight and 17.8% were obese, with a higher prevalence of obesity in the study group than in the control group (10.5%) (16). Interestingly, a study from Korea showed that fat intake, but not BMI, was associated with NSOM (11). Specifically, in the healthy weight group, higher fat intake was associated with a higher risk of NSOM. Unfortunately, our study did not determine whether BMI or fat contributed more to NSOM. More research may be needed to confirm this.

It is well known that adenoid hypertrophy leads to NSOM. One study (17) has shown that children with chronic NSOM have a higher incidence of overweight or obesity, and the presence and degree of adenoid or tonsil hypertrophy are not related to overweight or obesity, which indirectly suggests that tonsil and adenoid hypertrophy are not confounders between obesity and NSOM. Normal Eustachian tube function is the foundation for maintaining the normal function of the middle ear. Obese patients may alter the structure of the fat tissue around the Eustachian tube by altering its accumulation, thereby affecting Eustachian tube function and making NSOM more likely to occur (18). In addition, obesity is often associated with obstructive sleep apnea–hypopnea syndrome, which can lead to increased intra-abdominal pressure, decreased intrathoracic pressure, and exacerbation of gastroesophageal reflux (19). Reflux can cause damage to the mucosa of the Eustachian tube and tympanic cavity, thereby exacerbating the occurrence of NSOM. Therefore, changes in tube function and gastroesophageal reflux may be intermediate variables between obesity and NSOM.

For children, there is no gender difference in the incidence of NSOM, although NSOM is most common under the age of 2 and reaches another peak at the age of 5 (20). Gender and age are key factors for BMI (21, 22), which can be used to assess the greatest association between overweight and body fat and is widely used to measure obesity (23). To date, there is insufficient evidence for gender and age distribution differences in the higher BMI of NSOM patients than healthy children. Mehmet et al. (10) found that after grouping by gender, BMI remained statistically significantly higher in NSOM patients against controls in both boys and girls. They also found no difference in BMI between the NSOM and control groups at age (6, 8 for boys and 6, 9, 10, 11 for girls); however, the percentile range of BMI was higher in the NSOM group.

There are few reports about lipids related to NSOM. It has been reported that serum total cholesterol in the NSOM group is significantly higher than that in the controls, while triglycerides are not (15). However, another research has demonstrated that there is no difference in serum total cholesterol or triglycerides between the NSOM and control groups (16), which is consistent with our results. HDL-C and apoA1 were positive results for NSOM in this study. As HDL-C, LDL-C, and triglyceride have interaction relations, we put these three in a group for further MVMR analysis. ApoA1 is a major protein component of HDL (24), and apoB is a major protein component of LDL (25), and we put them in another group for MVMR. According to the MVMR analysis, the causal relationship between HDL-C and apoA1 on NSOM remained significant even after adjusting for the influence of other potential confounding factors on NSOM. We did not perform the MVMR analysis for obesity factors due to collinearity, nor did we perform the MVMR analysis of adipokines factors due to their independence.

In the two-step MR analysis for mediation, we used both BMI and obesity as exposures. Based on the analysis results, it was known that HDL-C and apoA can be used as mediating factors between obesity and NSOM. Combined with the direction of action indicated by the OR value, obesity/BMI may downregulate the production of HDL-C/apoA, and HDL-C/apoA1 plays a protective role in the development of NSOM. Therefore, obesity promotes NSOM by downregulating HDL-C/apoA1. Although the MR Egger method did not show consistent results with the other four methods when obesity was analyzed as an exposure and apoA1 as an outcome in MR analysis, the *p-*value of the MR Egger method, which was greater than 0.05, was considered not statistically significant. In addition to the IVW method, the p-value of the weighted median method was also less than 0.05, which enhanced the reliability of the results. Although there is no direct evidence that HDL-C/apoA1 is associated with NSOM, previous studies have found that HDL-C/apoA is associated with inflammation. The low levels of HDL-C are strongly associated with, and an independent predictor of, inflammation and endothelial cell activation (26). ApoA1 can play an anti-inflammatory role by inducing M2 macrophage differentiation (27) and inhibiting neutrophil hyperactivation (28). Obesity affects HDL-C in two ways. First, it accelerates HDL-C degradation, cholesteryl ester transfer protein (CETP) and hepatic lipase activity are elevated in obese patients. Increased hepatic lipase activity promotes HDL-C catabolism to produce apoA1 and HDL-C particles, with apoA1 being recycled or degraded by the kidneys. Second, it blocks HDL-C synthesis, which is also affected by CETP. CETP inhibitors block the exchange of triglycerides and cholesterol, reduce HDL-C esterification, and improve HDL-C function (29).

Among the adipokines, resistin and NGF showed a causal relationship with NSOM. We have not found any reports about resistin or NGF related to NSOM. Resistin was a risk factor for disease, and NGF was a protective factor. The MR analysis results had *p-*values only slightly lower than 0.05 and ORs close to 1, indicating that their significance was not high. We can only consider them as factors in a potential causal relationship with NSOM. Although the sensitivity analysis of “Leave-one-out” increased the reliability of the results, further validation is still needed.

There is also a view that NSOM may lead to obesity by affecting the chorda tympani nerve, leading to changes in taste function and preference for a high-fat diet (30). In our MR analysis using NSOM as exposure and 16 factors of obesity and lipid metabolism as outcomes, no positive results were obtained, suggesting that NSOM does not directly lead to obesity and does not affect lipid metabolism.

Finally, we recommend that weight loss is a good option for NSOM patients associated with obesity, especially in children. Even for NSOM patients with normal BMI, it is necessary to avoid a high-fat diet. However, it may be a new way to treat NSOM by regulating HDL-C, apoA1, resistin, and NGF. This article reveals the relationship between obesity, lipid metabolism-related factors, and NSOM at the genetic level. Compared to observational studies, the MR analysis excluded environmental factors and clarified their causal relationships, resulting in relatively reliable results. However, this article still has certain limitations. For example, we selected GWAS datasets for adiponectin and leptin levels from a mixed population, while other GWAS datasets came from European populations, which may have potential heterogeneity. To obtain causal factors related to NSOM as much as possible to facilitate the screening of mediators, we did not correct the *p-*value of the MR analysis results, which resulted in an increased false-positive rate of the results. Second, it was not possible to group datasets and obtain information on age and gender differences; however, previous studies have mostly focused on children. Additionally, a reverse MR analysis only provided insufficient lateral evidence and did not directly validate the viewpoint in the observational study that NSOM exacerbates obesity by affecting the sense of smell. Further prospective clinical trials are needed for us to remedy these limitations and validate our results.

## Conclusion

This study proposed several protective and risk factors related to obesity and lipid metabolism causality associated with NSOM. Through comprehensive analysis, we conclude that obesity and adiposity may increase the risk of developing NSOM while NSOM does not affect obesity, adiposity, or lipid metabolism. HDL-C and apoA may inhibit the progress of NSOM. In the adipokines, resistin may be a potential risk factor for NSOM, whereas NGF may be a potential protective factor. In addition, obesity may promote the development of NSOM by lowering HDL-C and apoA.

## Data availability statement

The original contributions presented in the study are included in the article/Supplementary material, further inquiries can be directed to the corresponding author.

## Ethics statement

The studies involving humans were approved by the Ethics Committee of Shaoxing People’s Hospital. The studies were conducted in accordance with the local legislation and institutional requirements. Written informed consent for participation was not required from the participants or the participants’ legal guardians/next of kin because The datasets were obtained from the IEU Open GWAS project, which is a public database.

## Author contributions

XY: Writing – original draft, Software, Investigation. SC: Writing – review & editing, Formal analysis, Data curation.
