# Supplementary material for: Obesity and adiposity promote the development of non-suppurative otitis media: a Mendelian randomization study
Source: Front Med (Lausanne). 2024 Jul 10;11:1422786. doi: 10.3389/fmed.2024.1422786 (PMC11266012; doi:10.3389/fmed.2024.1422786)
Supplement: Supplementary file 1 [file Table_1.docx]

**Table S1.** Reverse Mendelian randomization analysis results

| **exposure** | **outcome** | **method** | **nsnp** | **pval** | **OR(95%CI)** | **Egger_intercept_P** | **CochranQ_test_Pval** |
| --- | --- | --- | --- | --- | --- | --- | --- |
| NSOM | Obesity | IVW | 10 | 0.529587 | 0.95(0.81 - 1.12) |  | 0.073 |
| NSOM | Obesity | MR Egger | 10 | 0.361762 | 0.83(0.56 - 1.21) | 0.458 | 0.056 |
| NSOM | Body mass index | IVW | 10 | 0.098692 | 1.02(1.00 - 1.04) |  | 0.997 |
| NSOM | Body mass index | MR Egger | 10 | 0.328459 | 1.03(0.98 - 1.07) | 0.722 | 0.994 |
| NSOM | Body fat percentage | IVW | 16 | 0.084659 | 1.01(1.00 - 1.03) |  | ＜0.001 |
| NSOM | Body fat percentage | MR Egger | 16 | 0.611295 | 0.99(0.95 - 1.03) | 0.206 | ＜0.001 |
| NSOM | Waist circumference | IVW | 15 | 0.656993 | 1.00(0.99 - 1.02) |  | ＜0.001 |
| NSOM | Waist circumference | MR Egger | 15 | 0.426961 | 0.98(0.94 - 1.02) | 0.293 | ＜0.001 |
| NSOM | Hip circumference | IVW | 11 | 0.079843 | 1.03(1.00 - 1.06) |  | 0.512 |
| NSOM | Hip circumference | MR Egger | 11 | 0.609492 | 1.02(0.95 - 1.09) | 0.848 | 0.421 |
| NSOM | Total cholesterol | IVW | 6 | 0.503522 | 0.99(0.98 - 1.01) |  | 0.802 |
| NSOM | Total cholesterol | MR Egger | 6 | 0.693806 | 0.99(0.95 - 1.03) | 0.854 | 0.682 |
| NSOM | LDL cholesterol | IVW | 16 | 0.274698 | 1.01(0.99 - 1.02) |  | 0.073 |
| NSOM | LDL cholesterol | MR Egger | 16 | 0.431672 | 1.01(0.98 - 1.04) | 0.679 | 0.056 |
| NSOM | HDL cholesterol | IVW | 9 | 0.094961 | 0.96(0.92 - 1.01) |  | 0.017 |
| NSOM | HDL cholesterol | MR Egger | 9 | 0.102831 | 0.91(0.83 - 1.00) | 0.259 | 0.033 |
| NSOM | Triglycerides | IVW | 16 | 0.224785 | 1.01(0.99 - 1.03) |  | ＜0.001 |
| NSOM | Triglycerides | MR Egger | 16 | 0.984296 | 1.00(0.96 - 1.04) | 0.637 | ＜0.001 |
| NSOM | Apolipoprotein A1 | IVW | 16 | 0.512683 | 0.99(0.98 - 1.01) |  | 0.481 |
| NSOM | Apolipoprotein A1 | MR Egger | 16 | 0.975677 | 1.00(0.96 - 1.04) | 0.811 | 0.411 |
| NSOM | Apolipoprotein B | IVW | 6 | 0.426703 | 0.99(0.97 - 1.01) |  | 0.058 |
| NSOM | Apolipoprotein B | MR Egger | 6 | 0.526097 | 0.98(0.92 - 1.04) | 0.667 | 0.038 |
| NSOM | Adiponectin | IVW | 11 | 0.756177 | 1.00(0.98 - 1.03) |  | 0.197 |
| NSOM | Adiponectin | MR Egger | 11 | 0.258493 | 1.04(0.97 - 1.12) | 0.269 | 0.231 |
| NSOM | Resistin | IVW | 16 | 0.263441 | 0.98(0.94 - 1.02) |  | 0.609 |
| NSOM | Resistin | MR Egger | 16 | 0.791036 | 1.02(0.91 - 1.14) | 0.470 | 0.577 |
| NSOM | leptin | IVW | 4 | 0.868922 | 0.99(0.91 - 1.08) |  | 0.124 |
| NSOM | leptin | MR Egger | 4 | 0.232871 | 1.14(0.98 - 1.32) | 0.191 | 0.375 |
| NSOM | Agouti-related protein | IVW | 16 | 0.654831 | 0.98(0.92 - 1.05) |  | 0.008 |
| NSOM | Agouti-related protein | MR Egger | 16 | 0.570231 | 1.05(0.89 - 1.25) | 0.470 | 0.008 |
| NSOM | Nerve grows factor | IVW | 11 | 0.334726 | 0.93(0.80 - 1.08) |  | 0.073 |
| NSOM | Nerve grows factor | MR Egger | 11 | 0.533021 | 0.87(0.58 - 1.31) | 0.755 | 0.056 |

nsnp: number of single nucleotide polymorphisms used as instrumental variables for Mendelian randomization; pval: vaule of *p*; OR: odds radio; CI:confidence interval; NSOM: nonsuppurative otitis media; IVW: inverse-variance weighted.
