# Supplementary material for: Obesity and adiposity promote the development of non-suppurative otitis media: a Mendelian randomization study
Source: Front Med (Lausanne). 2024 Jul 10;11:1422786. doi: 10.3389/fmed.2024.1422786 (PMC11266012; doi:10.3389/fmed.2024.1422786)
Supplement: Supplementary file 2 [file Table_2.docx]

**Table S2.** P-value of Egger intercept in two-step MR for mediation.

| **interaction relationship** | **exposure** | **outcome** | ***p*-value** |
| --- | --- | --- | --- |
| Obesity- HDL cholesterol- NSOM | Obesity | HDL cholesterol | 0.891 |
|  | HDL cholesterol | NSOM | 0.502 |
| Obesity- Apolipoprotein A1- NSOM | Obesity | Apolipoprotein A1 | 0.284 |
|  | Apolipoprotein A1 | NSOM | 0.972 |
| Body mass index- HDL cholesterol- NSOM | Body mass index | HDL cholesterol | 0.789 |
|  | HDL cholesterol | NSOM | 0.502 |
| Body mass index- Apolipoprotein A1- NSOM | Body mass index | Apolipoprotein A1 | 0.651 |
|  | Apolipoprotein A1 | NSOM | 0.971 |

NSOM: nonsuppurative otitis media; IVW: inverse-variance weighted.
